# Organisation of extracellular matrix proteins laminin and agrin in pericapillary basal laminae in mouse brain

**DOI:** 10.1007/s00429-020-02036-3

**Published:** 2020-02-18

**Authors:** Eystein Hellstrøm Hoddevik, Shreyas Balachandra Rao, Soulmaz Zahl, Henning Bünsow Boldt, Ole Petter Ottersen, Mahmood Amiry-Moghaddam

**Affiliations:** 1 Division of Anatomy, Department of Molecular Medicine, Institute of Basic Medical Sciences, University of Oslo, Blindern, Post box 1105, 0317 Oslo, Norway; 2 Department of Pathology, Oslo University Hospital, Oslo, Norway; 3 Present Address: Department of Pathology, Odense University Hospital, Odense, Denmark; 4 Present Address: President’s Office, Karolinska Institutet, Nobels väg 6, 171 77 Stockholm, Sweden

**Keywords:** Extracellular matrix, Basal lamina, Heterogeneity, Agrin, Laminin, Astrocytes, Pericytes, α-Syntrophin, Aquaporin-4

## Abstract

Evidence suggests that extracellular matrix molecules of perivascular basal laminae help orchestrate the molecular assemblies at the gliovascular interface. Specifically, laminin and agrin are thought to tether the dystrophin-associated protein (DAP) complex to the astrocytic basal lamina. This complex includes α-syntrophin (α-Syn), which is believed to anchor aquaporin-4 (AQP4) to astrocytic endfoot membrane domains. We have previously shown that the size of the perivascular AQP4 pool differs considerably between brain regions in an α-Syn-dependent manner. Also, both AQP4 and α-Syn occur at higher densities in endfoot membrane domains facing pericytes than in endfoot membrane domains facing endothelial cells. The heterogeneous distribution of AQP4 at the regional and capillary level has been attributed to a direct interaction between AQP4 and α-Syn. This would be challenged (1) if the microdistributions of laminin and agrin fail to align with those of DAP and AQP4 and (2) if targeted deletion of α-Syn leads to a loss of laminin and/or agrin. Here, we provide the first detailed and quantitative analysis of laminin and agrin in brain basal laminae of mice. We show that the microdistributions of these molecules vary in a fashion that is well aligned with the previously reported microdistribution of AQP4. We also demonstrate that the expression patterns of laminin and agrin are insensitive to targeted deletion of α-Syn, suggesting that α-Syn deletion affects AQP4 directly and not indirectly via laminin or agrin. These data fill remaining voids in the current model of how key molecules are assembled and tethered at the gliovascular interface.

**Electronic supplementary material:**

The online version of this article (10.1007/s00429-020-02036-3) contains supplementary material, which is available to authorized users.

## Introduction

Transport and signalling mechanisms at the gliovascular interface are critically involved in a number of brain functions (Attwell et al. 2010; Iliff et al. 2012; Mishra 2017; Nagelhus and Ottersen 2013; Verkhratsky and Nedergaard 2018; Xie et al. 2013). In the past decade, much interest has been devoted to the water channel aquaporin-4 AQP4, which is abundantly expressed in astrocyte endfoot membranes. Targeted deletion of this channel or of its anchoring molecule, α-syntrophin (α-Syn), protects against brain oedema formation (Amiry-Moghaddam et al. 2003; Manley et al. 2000) and there is an ongoing debate as to what extent AQP4 is involved in facilitating clearance of waste products from brain neuropil (Iliff et al. 2012; Smith et al. 2017; Xie et al. 2013). Further, a mislocalisation of AQP4 in endfoot membranes has been demonstrated in several neurological conditions (Eid et al. 2005; Frydenlund et al. 2006; Ren et al. 2013; Sinclair et al. 2007; Yang et al. 2011) and precedes the development of chronic seizures in an animal model of temporal lobe epilepsy (Alvestad et al. 2013). These experimental data have prompted us to investigate the molecular mechanisms that regulate the expression of AQP4 in astrocyte endfeet at the gliovascular interface.

The expression of endfoot AQP4 is orchestrated by the dystrophin-associated protein (DAP) complex (Frigeri et al. 2001; Neely et al. 2001; Nico et al. 2003; Vajda et al. 2002). Evidence suggests that dystrophin anchors AQP4 through α-Syn and connects to the endothelial basal lamina through α- and β-dystroglycan (Neely et al. 2001). These molecular interactions likely explain the highly specific accumulation of AQP4 in perivascular endfoot membranes. A key piece of evidence in this scheme is our finding that the size of the perivascular AQP4 pool displays a significant regional heterogeneity and that *α-Syn* deletion removes a substantial and fairly constant proportion of AQP4 from perivascular endfoot membranes across brain regions. More specifically, between 79 and 94% of the endfoot pool of AQP4 was lost following deletion of the gene encoding α-Syn, with the low and high extremes represented by spinal cord and neocortex, respectively (Hoddevik et al. 2017). Moreover, both AQP4 and α-Syn occur at higher densities in endfoot membrane domains facing pericytes than in endfoot membrane domains facing endothelial cells. We concluded that α-Syn, through its interaction with AQP4, is the single most important factor determining the size of the perivascular AQP4 pool and hence an important regulator of the capacity for water transport and (putatively) for waste clearance.

Our conclusion must be tempered by the possibility that *α-Syn* deletion impacts AQP4 indirectly—through other molecules that regulate AQP4 expression—rather than only through a direct interaction with AQP4. Specifically, the question arises whether removal of *α-Syn* affects the distribution or concentration of the basal lamina proteins laminin and agrin—the very molecules that serve to tether the DAP complex and AQP4 to the astrocytic endfoot membrane.

Further, laminin and agrin bind to dystroglycan (DG) (Gesemann et al.^1996^, ^1998^; Michele et al. 2002) and were shown to induce the expression of AQP4 in a tailor-made model system (Camassa et al. 2015). The distribution of AQP4 is associated with lipid rafts and evidence has been provided for an interdependence between DG and laminin, whereby DG-associated proteins reorganise upon treatment with laminin (Guadagno and Moukhles 2004; Noel et al. 2009; Tham et al. 2016). Also, these basal lamina proteins are the first to appear in postnatal development (Lunde et al. 2015) and are implicated in the polarised expression of AQP4 in astrocytes and the formation of AQP4 supramolecular assemblies (Fallier-Becker et al. 2011; Noell et al. 2007, 2009).

The aim of this study was twofold. First, given the important instructive roles of agrin and laminin (Camassa et al. 2015; Fallier-Becker et al. 2011; Guadagno and Moukhles 2004; Lunde et al. 2015; Noell et al. 2009), we set out to unravel the modes of expression of these molecules in pericapillary basal laminae of brain. To the best of our knowledge, this is the first quantitative and detailed analysis using anti-agrin and anti-pan-laminin antibodies in mouse central nervous system at the ultrastructural level. Second, we used quantitative immunogold histochemistry to assess whether targeted deletion of *α-Syn*—a key organiser of proteins at the blood–brain interface—affects the level or microlocalisation of laminin or agrin. If so, this would challenge the idea that *α-Syn* affects AQP4 expression solely through its well-documented role as an AQP4 anchor.

## Methods

### Animals

We used adult male C57BL/6 mice (Jackson Laboratories, Boulder, CO), which served as wild-type (WT) controls, and adult male *Snta1* knockout (*α-Syn*−/−) mice. The latter, transgenic strain was generated as described previously (Adams et al. 2000). Mice were allowed ad libitum access to food and drinking water. Animal experiments were performed according to the European Council law on protection of laboratory animals, with the approval of the University of Oslo’s Animal Care and Use Committee (FOTS-12077 and FOTS-2744). Every effort was made to minimise the number of animals.

### Antibodies

Anti-agrin labelling was performed using a polyclonal rabbit antibody received as a kind gift from Professor Markus A. Ruegg, University of Basel. The primary antibody solution was diluted 1:500 for immunogold labelling and 1:1000 for immunoperoxidase staining. Anti-laminin labelling was done using a rabbit polyclonal antibody (Sigma-Aldrich L9393) diluted 1:100 for immunoperoxidase and immunogold labelling.

Visualisation of anti-agrin and anti-laminin staining for electron microscopy was done using a goat anti-rabbit antibody conjugated with 15-nm colloidal gold particles (Abcam) and diluted 1:20. For immunoperoxidase staining, a biotinylated donkey anti-rabbit (Pierce) secondary antibody diluted 1:100 was used for both anti-agrin and anti-laminin experiments.

The primary antibodies used for immunofluorescence were anti-β-dystroglycan (1:100 dilution; beta-dystroglycan (H-242) antibody, Santa Cruz Biotechnology; Cat# sc-28535; RRID:AB_782259) and anti-AQP4 (1:400 dilution; Sigma Aldrich; Cat# A5971; RRID:AB_258270). Cy3 donkey anti-rabbit (Jackson ImmunoRe-search Labs; Cat#: 711-165-152; RRID:AB_2307443) was used as secondary antibody in a 1:500 dilution. Vessels were stained using DyLight^®^ 649 conjugated tomato lectin (LEL, TL; Vector labs; Cat#: DL-1178).

### Post-embedding immunogold electron microscopy

Mice were anaesthetised with a single intraperitoneal injection of equithesin (5 μL/gram body weight) and transcardial perfusion fixation was performed according to a pH-shift protocol as previously described (Promeneur et al. 2013), so that one-half of each brain could be used for light microscopy, while the other half was used for electron microscopy. For electron microscopy brain hemispheres (*n* = 4 per group) were cut into 0.5–1.0 mm slices, regions were dissected, cryoprotected, quick frozen in liquid propane (− 170 °C), and subjected to freeze substitution. Specimens obtained from cerebellum (CB), cerebral cortex (CX) and optic nerve (ON) were embedded. The latter region was chosen to study the meningeal covering. In contrast to other brain regions, embedding in resins of cross-sectioned optic nerve leaves the surrounding meninges attached, probably due to the circular envelopment. All specimens were embedded in methacrylate resin (Lowicryl HM20) and polymerised by UV light below 0 °C. Ultrathin sections (70–100 nm) were cut using an Ultratome (Reichert Ultracut S, Leica) and placed on 300 mesh grids.

Immunogold labelling was carried out as previously described (Lunde et al. 2015). Briefly, sections were rinsed in Tris-buffered saline with Triton X-100 (TBS-T; 5 mM Tris–HCl, 0.3% NaCl, 0.1% Triton X-100), incubated in 2% human serum albumin (HSA), followed by primary antibody (anti-agrin or anti-laminin) overnight, secondary antibody (15 nm gold) for 90 min, and contrasted with 2% uranyl acetate for 90 s and 0.3% lead citrate for 90 s. Sections were examined using a Tecnai 12 electron microscope at 80 kV. The examiner was blinded for animal genotype. Primary antibody was omitted on control sections.

### Immunoperoxidase staining

Immunohistochemistry and antigen retrieval with pepsin digestion of thick sections were performed as previously described (Franciosi et al. 2007; Lunde et al. 2015). Antigen retrieval steps with altered pH (citric acid) were attempted without noticeable change from control experiments. All sections were, therefore, treated with pepsin prior to incubation with primary antibodies.

### Immunofluorescence staining

WT and *α-Syn−/−* mice were deeply anesthetised and then decapitated. Brains were subsequently removed quickly from the cranium, placed in OCT medium and cryomold cassettes, and then immediately frozen in liquid nitrogen. Sections were cut 14-µm thick using a cryostat, adhered onto glass slides and stored at − 80 °C until use. Prior to staining, sections were thawed to room temperature and fixed using 2% formaldehyde for 15 min. Immunofluorescence experiments were performed as previously described (Rao et al. 2019). Images of neocortex and cerebellum were acquired using LSM 710 confocal microscope at 20× magnification (Carl Zeiss). Identical settings were used when acquiring images from both experimental groups.

### Immunogold quantitation

Quantitative analysis was performed as previously described (Hoddevik et al. 2017). Briefly, images of 20–30 capillaries were acquired from each subregion present on each section. Images were acquired so that a similar length of astrocyte membrane adjacent to endothelium and pericytes was shown on each picture. Inclusion and exclusion criteria were defined for capillaries, astrocyte endfeet and pericytes prior to image collection. Pericytes were defined as perivascular cells surrounded by a clearly defined ad- and abluminal basal lamina. An arbitrary line, drawn in the middle of the basal lamina and thus equidistant from neighbouring cell membranes, was used to quantify linear density of gold particles for anti-agrin and anti-laminin. Histograms of gold particle distribution in the basal lamina abutting astrocytes were determined along an axis perpendicular to the midline described above. Gold particles were included in a region of interest (ROI) sufficiently large to accommodate for the theoretical distance between gold particle and epitope (Amiry-Moghaddam and Ottersen 2013). Linear densities of gold particles in capillary basal lamina were determined by an extension of analysis [Soft Imaging Systems (SIS), Münster, Germany]. Linear densities were determined semi-automatically and transferred to SPSS Version 22 (SPSS, Chicago, IL, USA) for statistical analysis. When comparing CX labelling to that of CB, images from all subregions were included (CB-mol, CB-gran and CB-white) and only basal lamina domains abutting astrocytes were included. No antigen recovery was needed for immunogold experiments and only basal lamina labelling was quantified.

Comparisons between groups were made by one-way ANOVA with post hoc Scheffe tests, Student’s *t* test and confirmed by non-parametric Mann–Whitney *U* test. Data are presented as mean ± standard error of the mean (SEM).

### Quantitative RT-PCR

Mice (*n* = 4 for each genotype) were anaesthetised with isofluorane and decapitated followed by immediate removal of the brain from the cranium. Regional dissections (*n* = 4) of CX and CB were processed for quantitative RT-PCR analysis by overnight incubation in RNAlater (Ambion) and storage at − 80 °C until further processing. Quantitative RT-PCR was carried out as previously described (Hoddevik et al. 2017). We used the following primers: 5′-CAGTGGGGGACCTAGAAACA-3′ (sense) and 5′-ATGGCCAGAGCCATGTAGTC-3′ (antisense) for agrin (Agrn, exon boundary 33–34), 5′-TGGATAAAGACAGGCCCTTG-3′ (sense) and 5′-ACTTTGGCACTGCTGATTCC-3′ (antisense) for laminin α1 (*Lama1,* exon boundary 60–61), 5′-ACCAGCCTACCTCCAGCTTT-3′ (sense) and 5′-CCCATTCCATCCATCTTCTG-3′ (antisense) for laminin α2 (*Lama2,* exon boundary 62–63) and 5′-ACGGACAACTGCGTTGATTT-3′ (sense) and 5′-CAAGGCCTTCCAGCCTTATAG-3′ (antisense) for TATA-box binding protein (*Tbp, exon boundary* 5–6).

Statistical analysis was performed by one-way ANOVA, post hoc Scheffe test and Student’s *t* test. Bootstrapping was used to calculate confidence intervals. Standard deviations prior to bootstrapping are also shown. TATA-box binding protein (*Tbp*) was used as the normalisation gene. Data are presented as mean with 95% confidence intervals calculated by bootstrapping.

## Results

### Agrin and laminin are expressed in pericapillary basal laminae and at the glia limitans

**Fig. 1:**
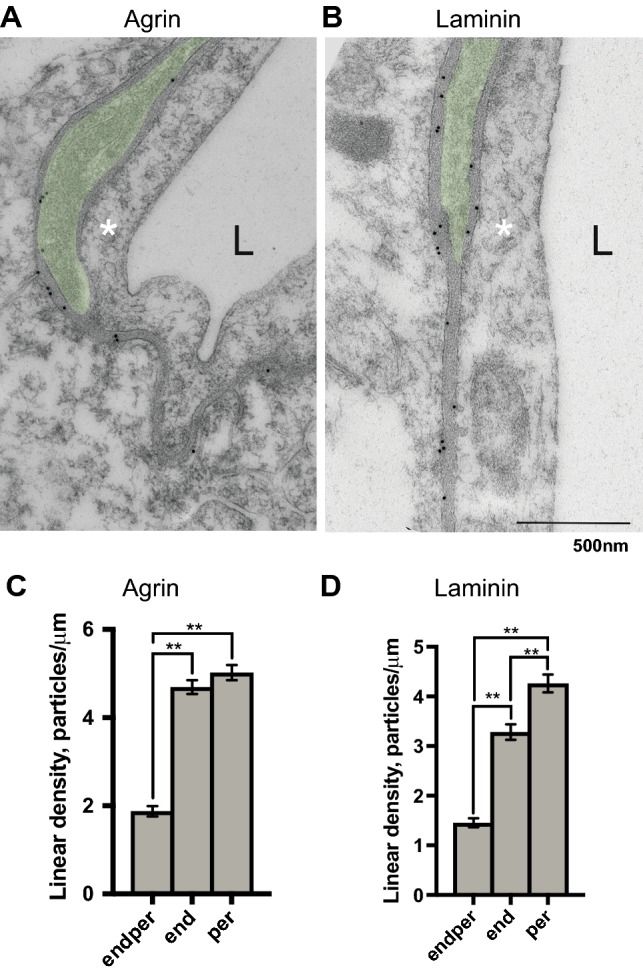
Evidence of heterogeneous labelling intensity of basal lamina microdomains around capillary bed for agrin and laminin is shown. **a**, **b** Representative electron micrographic images of anti-agrin and anti-laminin immunogold labelling around capillary bed. Endothelium (annotated with asterisk), capillary lumen (L) and part of a pericyte (coloured green), surrounded by a basal lamina on both sides are shown. Astrocyte endfeet abut the basal lamina overlying both pericytes and endothelium. Immunogold particles directed against agrin (**a**), were quantified as gold particles per unit length (μm) for all of the depicted basal lamina microdomains (**c**); between astrocytes and endothelium (end mean = 4.692, Std error = 0.158), between astrocytes and pericytes (per mean = 5.022, Std error = 0.172) and between pericytes and endothelium (*endper* mean = 1.878, Std error = 0.117). Labelling intensity differs depending on the adjoining cell type and is notably more intense in the two basal lamina microdomains abutting astrocytes compared with that interlaced between pericytes and endothelium. This difference (linear density, graph **c**) is statistically significant (*p* < 0.001). No difference is found between the end and per microdomains. Immunogold labelling against laminin (**b**, **d**) displays a similar pattern (end mean = 3.282, Std error = 0.158; per mean = 4.263, Std error = 0.181; endper mean = 1.456, Std error = 0.092). Differences between all three basal lamina microdomains are statistically significant (graph **d**, *p* < 0.001) and labelling is most pronounced in the basal lamina interlaced between astrocyte endfeet and pericytes. Scalebar 500 nm. **Signifies *p* < 0.001. Error bars represent standard error of the mean (SEM)

Immunoperoxidase experiments on sections treated with pepsin for antigen retrieval, shown in supplementary Fig. 1 (S1) demonstrate labelling for laminin and agrin around vessels and at the glia limitans. Pepsin proteolysis exposes otherwise inaccessible epitopes, but may also generate new, unspecific epitopes. Without pepsin digestion, however, only faint labelling was seen for both anti-laminin and anti-agrin staining (not shown). No staining was observed when the primary antibodies were omitted (S1B, inset). Electron microscopic ultrathin sections (Fig. 1a, b) from different brain regions show distinct immunogold labelling in basal lamina between astrocyte endfeet and endothelium (*end*), between pericytes and endothelium (*endper*), and between astrocyte endfeet and pericytes (*per*). Both anti-laminin and anti-agrin immunoperoxidase staining on thick sections as well as immunogold labelling on ultrathin sections display unspecific labelling of nuclei.

### Heterogeneous microdistribution of laminin and agrin in pericapillary basal lamina domains

Quantitative immunogold analysis of laminin and agrin labelling in pericapillary basal lamina domains shows that the labelling intensity for both laminin and agrin depends on the adjoining cell type (Fig. 1a, b). Thus, labelling is more pronounced in the basal lamina domains facing astrocytes (*end, per*) than in those domains that are interposed between endothelium and pericytes (*endper)*. When quantified as number of gold particles per µm unit length of basal lamina, this difference is statistically significant (*p* < 0.001) for both proteins (Fig. 1c, d).

For laminin, there are statistically significant differences between all three basal lamina domains (Fig. 1d, *p* < 0.001). The basal lamina interposed between astrocyte endfeet and pericytes (*per*) shows more intense labelling than the basal lamina domain between astrocyte endfeet and endothelial cells (*end)*. For agrin, there is no significant difference between *end* and *per* portions of the basal lamina (Fig. 1c).

### Quantitative RT-PCR demonstrates significant regional differences for laminin α-2 and agrin mRNA when comparing cortex and cerebellum.

**Fig. 2:**
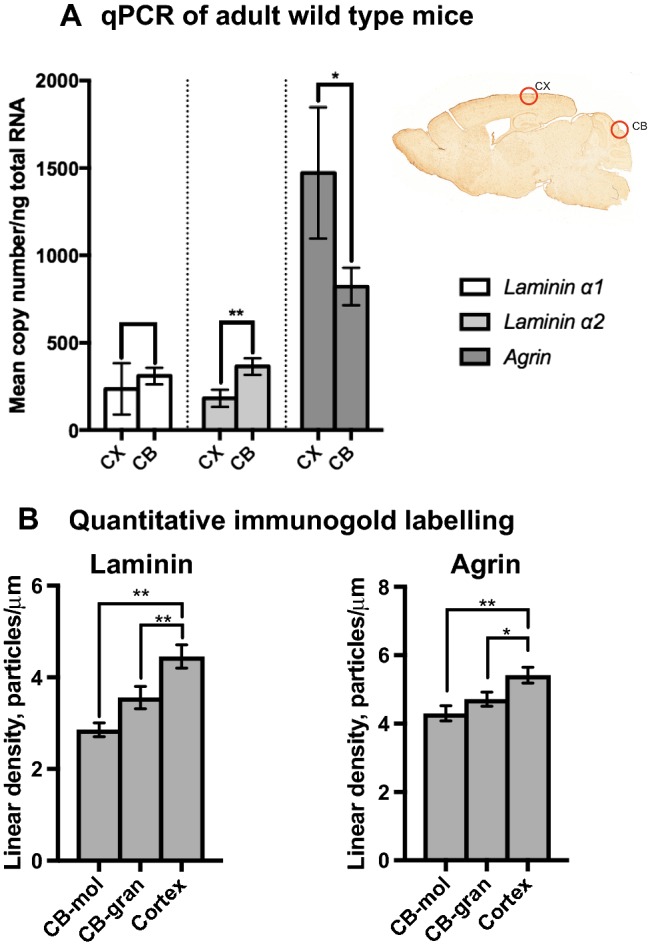
Evidence of regional heterogeneity of agrin and laminin is shown by quantitative RT-PCR (**a**) and immunogold labelling (**b**). Top right picture in **a** (right) shows dissected regions for PCR and embedded regions for electron microscopy. Graph **a** shows quantitative RT-PCR of ECM genes laminin (isoforms laminin α-1 and laminin α-2) and agrin in CX and CB from adult WT mice. All levels are reported as copy number/ng of total RNA. Levels of agrin mRNA are significantly higher (*p* = 0.015) in CX (mean 1471.91, SD 236.70) compared with CB (mean 821.81, SD 67.07). There is no significant difference (*p* = 0.238) between mRNA levels for *laminin α-1* when comparing CX (mean 235.77, SD 92.54) with CB (mean 309.96, SD 30.08). Levels of *laminin α-2* mRNA, however, are significantly (*p* < 0.001) higher in CB (mean 364.49; SD 29.89) compared with CX (mean 182.22; SD 31.41). Quantitative immunogold analysis of agrin (**b**, right) and laminin (**b**, left) in pericapillary basal laminae shows differences between brain regions. Using non-parametric Mann–Whitney *U* test, agrin levels in sublayers of the cerebellum, CB-mol (mean 4.3007; std error 0.21999) and CB-gran (mean 4.7148; std error 0.20698), show a statistically significant difference compared with those of CX (mean 5.4155; std error 0.22868), respectively *p* = 0.001 and *p* = 0.021. Laminin levels also vary across examined brain regions and subregions (left). Laminin levels in CX (mean 4.4523; std error 0.25444) are higher than in CB-mol (mean 2.8572; std error 0.14975) and CB-gran (mean 3.5607; std error 0.24393), respectively, *p* < 0.001 and *p* = 0.001. Error bars in **a** represent 95% confidence intervals calculated by bootstrapping. Error bars in **b** represent standard error of the mean (SEM). *p < 0.05, **p < 0.001

While immunoperoxidase labelling of either protein displays no obvious difference between different brain regions, such differences do occur at the mRNA level. We performed a quantitative analysis of *laminin α-1* (*LAMA1*)*, laminin α-2* (*LAMA2*) and *agrin* (*AGRN*) mRNA on samples obtained from CX and CB from wild-type (WT) mice (Fig. 2a). While no difference between CX and CB is detected for *laminin α-1* mRNA, there are statistically significant differences for *laminin α-2* and *agrin.* As illustrated, *agrin* mRNA levels are significantly higher in cerebral cortex than in cerebellum. The reverse is true for *laminin α-2* which is more abundant in the cerebellum than in the cerebral cortex.

### Laminin and agrin immunogold labelling at the pericapillary level and in the immediate proximity of glia limitans

Shown in Fig. 2b are quantitations of immunogold linear density for laminin (left) and agrin (right). Consistently, labelling intensity for both proteins is least pronounced around capillaries in the cerebellar molecular layer (CB-mol), followed by those in the cerebellar granular cell layer (CB-gran) and highest in cerebral cortex. There are statistically significant differences when comparing cerebellar sublayers to cerebral cortex for both proteins.

At the subpial surface, the labelling pattern of agrin (S3A) is similar to that of laminin (S3B). A linear array of immunogold particles is demonstrated for both proteins corresponding to the basal lamina apposed to the glia limitans.

### β-Dystroglycan remains unaltered following α-Syn gene deletion

**Fig. 3:**
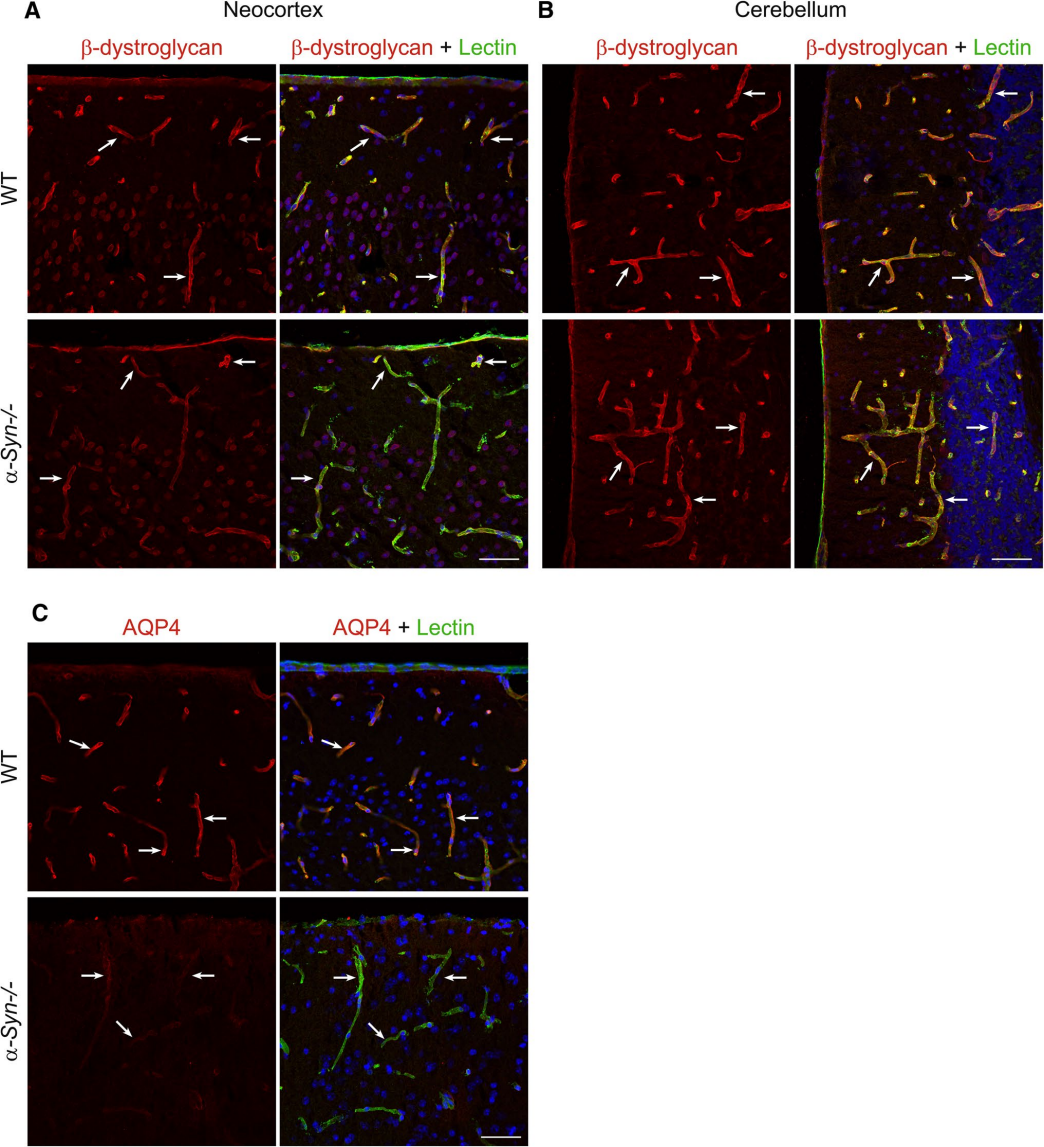
Immunofluorescence of anti-β-dystroglycan and anti-AQP4 staining in brain is shown. **a**, **b** demonstrate labelling of β-dystroglycan (red) and the endothelial marker lectin (green) in cortex (**a**) and cerebellum (**b**) of WT (top) and *α-Syn−/−* (bottom) mice. Anti-β-dystroglycan staining is seen in perivascular domains (white arrows) of WT mice and labelling remains unchanged following *α-Syn−/−* genetic knockout. Shown in **c** is anti-AQP4 labelling from cortex in both genotypes as internal control. Labelling on WT sections (top) yields an anticipated, strong, perivascular signal. This signal is markedly reduced in *α-Syn−/−* mice (bottom). Nuclear staining (DAPI) is shown in blue. Arrows indicate blood vessels. Scale bars = 50 µm

To examine whether *α-Syn* deletion affects β-dystroglycan (β-DG), which links the DAP complex to the basal lamina proteins, we studied localisation of this protein. Confocal immunofluorescence images in Fig. 3a, b show anti-β-dystroglycan (β-DG) labelling in WT vs *α-Syn−/−* mice. Distinct, perivascular labelling is seen in both examined regions (cortex and cerebellum) without any noticeable difference in labelling intensity or distribution when comparing genotypes.

### Regional differences in laminin and agrin gene transcription remain unaffected by α-Syn gene deletion

**Fig. 4:**
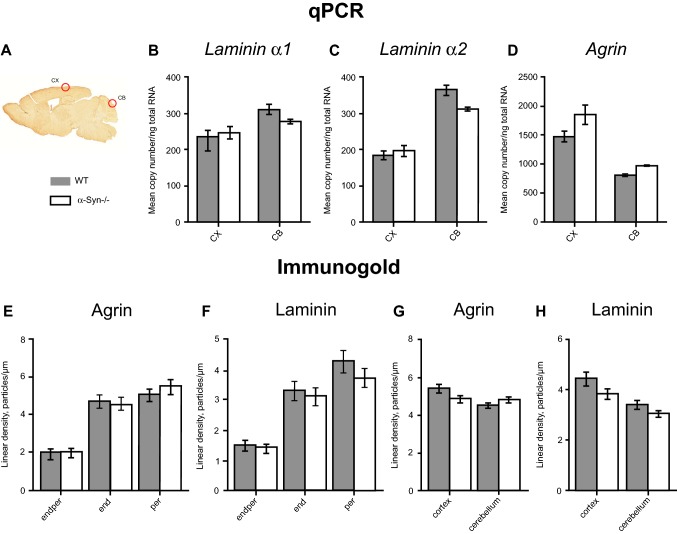
Shown in **a** is a sagittal section of adult mouse brain depicting examined regions (red circles): cerebral cortex (CX) and cerebellum (CB). Graphs **b**–**d** show quantitative RT-PCR of *laminin α-1*, *laminin α-2* and *agrin* mRNA (copy number/ng of total RNA) from CX and CB, and compare WT with *α-Syn*−/− genotypes. Analysis of *laminin α-1***b** demonstrates similar levels between CX and CB in WT mice (CX: mean 235.77, SD 92.54 and CB mean 309.96, SD 30.08) which remain unaltered following gene deletion of *α-Syn* (CX mean 246.53, SD 36.81; CB mean 277.40, SD 9.51). Data for *laminin α-2***c** demonstrate regional differences in WT controls (CX mean 182.22, SD 31.41; CB mean 364.49, SD 29.90) and similar differences are observed in *α-Syn−/− mice* (CX mean 196.00, SD 28.13 and CB mean 310.80, SD 6.55). Analysis of *agrin* demonstrates similar regional differences between CX (mean 1861.13; SD 435.95) and CB (mean 964.55; SD 37.76) in *α-Syn−/− mice* as seen in WT controls (CX: mean 1471.91; SD 236.70 and CB: mean 821.82; SD 67.06). Error bars in **b**–**d** represent 95% confidence intervals, calculated by bootstrapping. Figures **e**–**h** show quantitative immunogold labelling of agrin and laminin, measured as linear density of gold particles per µm length of the basal lamina. Data are shown as one single column for each basal lamina microdomain or anatomical region. Analysis for both proteins **e**, **f** shows no difference between genotypes when examining basal lamina microdomains (endper, end, per). At the regional level, the heterogeneous expression of both laminin and agrin in the basal lamina of the cortical and cerebellar capillary bed thus seems independent of *α-Syn*. Figures **g**, **h** depict quantitative immunogold labelling of capillary basal lamina: Images from sublayers of CB were merged (CB-mol, CB-gran and CB-white). The basal lamina enclosed between pericytes and endothelium (endper) was not included so as to assess influence of astrocytes upon agrin and laminin expression. The remaining basal lamina microdomains were merged as one datapoint per acquired image. Genetic deletion of *α-Syn* does not impact on the observed regional differences in immunogold labelling (agrin and laminin alike). No difference is found when comparing levels of either protein (agrin or laminin) in WT vs *α-Syn−*/− genotypes, neither for CX nor for CB. Error bars in **e**–**h** represent standard error of the mean (SEM)

Quantitative RT-PCR of *laminin α-1* (Fig. 4b) in *α-Syn* null mice is similar to what is observed in WT mice. The analysis for *laminin α-2* (Fig. 4c) shows regional differences in gene transcription that remain unaffected by *α-Syn* gene deletion. Similarly, analysis of *agrin* (Fig. 4d) demonstrates regional differences of similar magnitude in *α-Syn*−*/*− as in WT controls.

### Immunogold labelling of pericapillary basal lamina domains remains unaltered following α-Syn gene deletion

We performed a quantitative immunogold analysis using anti-laminin and anti-agrin antibodies. We compared labelling of cerebellum and cerebral cortex at the pericapillary level for *α-Syn*−/− mice and WT controls. Statistical analysis for both proteins using Mann–Whitney *U* test showed no difference between genotypes when comparing respective basal lamina domains (*endper, end, per*), nor any difference for the examined regions (Fig. 4e–h).

### Transversal histograms of pericapillary basal laminae demonstrate a normal distribution of anti-agrin and anti-laminin immunogold particles around the basal lamina midline

**Fig. 5:**
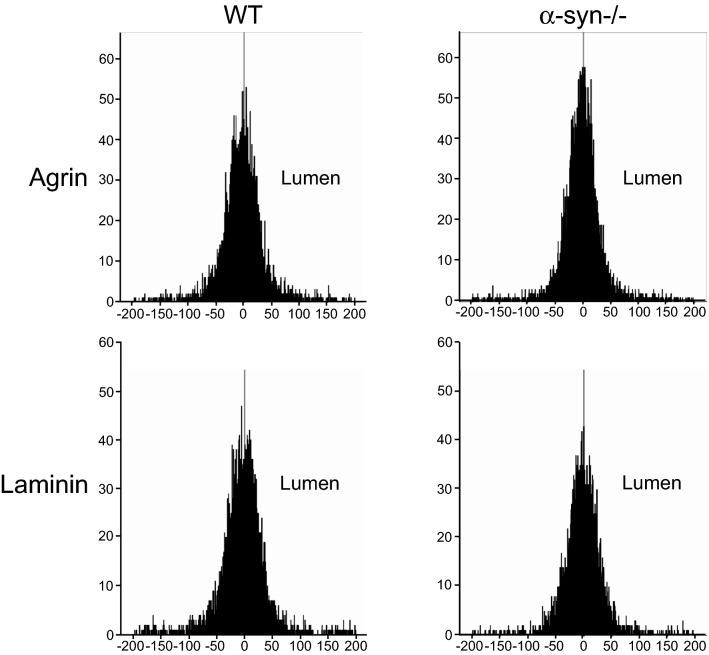
Impact of genetic deletion of *α-Syn* on agrin and laminin distribution within perivascular basal lamina abutting astrocytes is shown. Histograms demonstrate gold particle distribution along an axis perpendicular to the middle (grey, central line in each histogram) of pericapillary basal lamina microdomains abutting astrocytes. The side towards capillary lumen is indicated (lumen). Data from CX and CB were pooled, the endper basal lamina microdomain excluded from the analysis. Data are shown for anti-agrin and anti-laminin immunogold labelling on WT vs *α-Syn*−/− tissue. *Y*-axis shows number of gold particles detected (frequency), *X*-axis represents distance from midpoint of the basal lamina in nm. Gold particles signalling laminin and agrin were normally distributed across the lamina with a distinct peak corresponding to its midline. No difference is seen between WT and *α-Syn−/−* genotypes

Shown in Fig. 5 are histograms of gold particle distribution along an axis perpendicular to the pericapillary basal laminae. Data are shown for anti-agrin and anti-laminin immunogold labelling on WT vs *α-Syn*−/− tissue. The three ROI types (*per, end* and *endper)* are merged and data are pooled from cerebellum and cerebral cortex. In both mouse lines (*α-Syn*−/− and WT), gold particles are normally distributed around the basal lamina midline. Shown in supplementary Fig. 2 (S2) are histograms with individual representations of *per, end* and *endper* basal lamina microdomains. Despite differences in labelling intensity, particles were consistently symmetrically distributed with respect to the basal lamina midline. No difference was found between WT and *α-Syn*−/− mice.

## Discussion

The extracellular matrix proteins, laminin and agrin, have long been recognised as important structural components of basal laminae in brain (Thomsen et al. 2017). Only more recently has it become clear that these molecules serve additional roles and that they are essential for upholding the functional specialisation of the gliovascular interface (Fallier-Becker et al. 2011; Noel et al. 2019). Notably, it is now realised that the functional polarisation of astrocytes depends on these matrix proteins (Camassa et al. 2015; Lunde et al. 2015; Neely et al. 2001). The accumulation of the brain water channel AQP4 in perivascular endfeet—a hallmark of astrocyte polarisation—is contingent on laminin. Thus, AQP4 is retained in perivascular astrocyte membranes through anchoring via α-Syn to the DAP complex, which in turn is held in place through a dystroglycan bridge linked to laminin in the perivascular basal lamina (Neely et al. 2001). Evidence has accrued to suggest that laminin and agrin are instructive in the sense that they initiate the build-up of the specific molecular assemblies in perivascular endfeet (Fallier-Becker et al. 2011; Lunde et al. 2015; Noell et al. 2007, 2009, 2012; Sato et al. 2018; Tham et al. 2016; Warth et al. 2004; Wolburg et al. 2009).

Given the key roles of laminin and agrin, it is surprising that so little is known about their organisation and modes of expression. Any heterogeneity in their distribution, regionally or subregionally, is likely to be reflected in functional heterogeneities at the gliovascular interface. Here, we provide the first quantitative immunocytochemical analysis at ultrastructural level of laminin and agrin in mouse brain. We have previously reported immunogold labelling and western blots using the anti-laminin and anti-agrin antibodies (Lunde et al. 2015). Immunogold labelling is distinct over the basal lamina for both antibodies (Fig. 1), also see Lunde et al. 2015). The precise localisation of both anti-laminin and anti-agrin immunogold signals to the basal lamina suggests specific labelling. Moreover, the anti-agrin antibody was previously tested on agrin knockout tissue (Eusebio et al. 2003). No knockout tissue is available to test the specificity of the laminin antibody. However, using western blotting, we have previously shown that incubation of immunoblots with whole brain homogenates generates a major band at ≈ 200 kDa and two weaker bands at ≈ 400 kDa and ≈ 600–700 kDa (Lunde et al. 2015). This is in accordance with prior publications, where the 200 kDa band corresponds to the β- and ϒ-chain of laminin, while the 400 kDa band corresponds to the α1-chain (Zhang et al. 2007).

Our quantitative immunogold analyses reveal that gold particles signalling laminin and agrin were symmetrically distributed across the lamina with a distinct peak corresponding to its midline. There were striking differences between specific basal lamina domains. Notably, basal lamina domains abutting astrocyte endfeet contain significantly more agrin and laminin than basal lamina domains interposed between endothelium and pericytes. Importantly, laminin occurs in higher density in the basal lamina compartments between the astrocyte endfeet and pericytes compared to those between the astrocyte endfeet and endothelial cells. This correlates with reported differences in AQP4 distribution (Gundersen et al. 2013; Hoddevik et al. 2017) and is compatible with an instructive role of laminin as previously suggested (Gautam et al. 2019; Yao et al. 2014).

The reported experiments were not specifically designed to identify the sites of laminin and agrin synthesis. We have previously shown astrocyte-conditioned medium to contain both proteins (Camassa et al. 2015). Boulay et al. (2017) recently analysed the endfeet transcriptome which was shown to contain α1 laminin, in addition to mRNA encoding for several other ECM genes (including collagen type XII α1, collagen type VI α5). Agrin was not detected. Current voids in our understanding of laminin and agrin mRNA synthesis do not detract from the conclusions of this paper as our results merely depend on the level and location of these molecules, not on their site of synthesis.

While the measured levels of basal lamina proteins—especially that of laminin—in different basal lamina microdomains are well aligned with that of AQP4, a similar alignment was not observed at the regional level when comparing cortex with cerebellum. Thus, the present immunogold data indicate that lamin and agrin are expressed at higher densities in cortex than in cerebellum. This contrasts with our data on AQP4 (Hoddevik et al. 2017) which showed higher labelling densities in the cerebellum than in cortex. This might indicate that the regulatory mechanisms governing expression levels of AQP4 and ECM proteins at the basal lamina microdomain level are different from mechanisms responsible for the regional heterogeneity of these proteins.

An important aim of the present study was to assess whether the expression of laminin and agrin is sensitive to deletion of *α-syntrophin*. If so, this would challenge the conclusion of our previous study (Hoddevik et al. 2017), that the loss of AQP4 following *α-Syn* deletion is explained in full by the direct interaction between α-Syn and AQP4. Given the instructive role of laminin and agrin (Lunde et al. 2015), any downregulation of these molecules after *α*-*Syn* deletion could confound the effect on AQP anchoring. Indeed, earlier studies point to a complex interdependence between the various molecules associated with the DAP complex (Nico et al. 2010; Eilert-Olsen et al. 2012; Nagelhus and Ottersen 2013). Our data indicate that *α-Syn* deletion has no effect on the expression of laminin or agrin at the mRNA level, nor on the expression and localisation of the two basal lamina proteins. We also show that expression and localisation of β-dystroglycan, the link between the DAP complex and the basal lamina proteins, remains unaltered following *α-Syn* deletion. Thus our conclusion (Hoddevik et al. 2017) holds: α-syntrophin is likely to dictate the expression level of AQP4 in endfoot membranes through its direct coupling to this water channel, rather than indirectly, by modulating the expression level of those matrix molecules that tether the DAP complex to endfoot membranes.

## Conclusion

Data from WT mice reveal significant differences between basal lamina microdomains when it comes to agrin and laminin expression. Differences correlate well with previously recorded microdistributions of AQP4 and α-Syn. Notably, high laminin levels in the proximity of pericytes may explain how AQP4 levels are higher in adjoining astrocyte endfoot membrane domains. Thus, in line with an instructive role of laminin, a higher concentration of this molecule would retain a higher number of DAP complexes and AQP4 molecules in adjoining membranes. Overall, findings are consistent with the idea that ECM proteins, agrin and laminin, enable membrane compartmentalisation in astrocyte endfeet, which in turn contributes to the polarised expression of AQP4 among other dystrophin-associated proteins. Targeted deletion of *α-Syn* leaves laminin and agrin distribution unaltered. This supports the hypothesis that loss of α-Syn affects AQP4 redistribution directly rather than indirectly via altered levels of the ECM proteins agrin and laminin.

## Electronic supplementary material

Below is the link to the electronic supplementary material.

Supplementary figure 1: Immunoperoxidase labelling of agrin (A) and laminin (B) is shown on sagittal sections from an adult mouse brain. Insets show higher magnification from cerebellum (CB) and cortex (CX). Higher magnification images are arbitrarily orientated with brain surface upwards. Both antibodies display a similar staining pattern whereby a distinct brim surrounds all vessels (arrows), consistent with labelling of basal lamina. Staining of brain surface and meningeal covering (insets and overview, arrowheads) is also present. Omitting primary antibodies (left, Neg control, inset) abolishes all labelling. Nuclear and Purkinje cell (inset, CB) staining is interpreted as unspecific. (PDF 9839 kb)

Supplementary figure 2: Histograms of individual basal lamina microdomains *per, end *and* endper* are shown. Gold particle distribution along an axis perpendicular to the middle (grey, central line in each histogram) of pericapillary basal lamina is shown for WT and *α-Syn*-/- genotypes. The capillary lumen is to the right in each graph. Datapoints from CX and CB were pooled. A: Anti-agrin immunogold labelling. B: Anti-laminin immunogold labelling. Gold particles signalling laminin and agrin were normally distributed across the lamina with a distinct peak corresponding to its midline. No difference is seen between WT and *α-Syn-/- *genotypes nor is any difference in distribution present when comparing individual ROI types with each other. Y-axis shows number of gold particles detected (frequency), X-axis represents distance from midpoint of the basal lamina in nm. (PDF 600 kb)

Supplementary figure 3: Immunogold labelling of agrin (A) and anti-laminin (B) adjacent to the subpial glia limitans from optic nerve is shown. The glial limiting membrane consists of astrocytes laden with glial fibrillary acidic protein (annotated with asterisk). Shown in both A and B is the tightly woven meningeal covering with collagen fibres cut in cross-section and in parallel. Gold particles conjugated to both laminin and agrin are concentrated in the basal lamina apposed to the outermost part of the glia limitans (arrows). Insets in both A and B show higher resolution of the region of interest. Scalebar in inset (B) 100 nm, scalebar in picture (B, right) 200 nm. (PDF 1153 kb)
