# Supplementary figures and images for: Organisation of extracellular matrix proteins laminin and agrin in pericapillary basal laminae in mouse brain
Source: Brain Struct Funct. 2020 Feb 18;225(2):805–16. doi: 10.1007/s00429-020-02036-3 (PMC7046580; doi:10.1007/s00429-020-02036-3)

**A: Agrin**

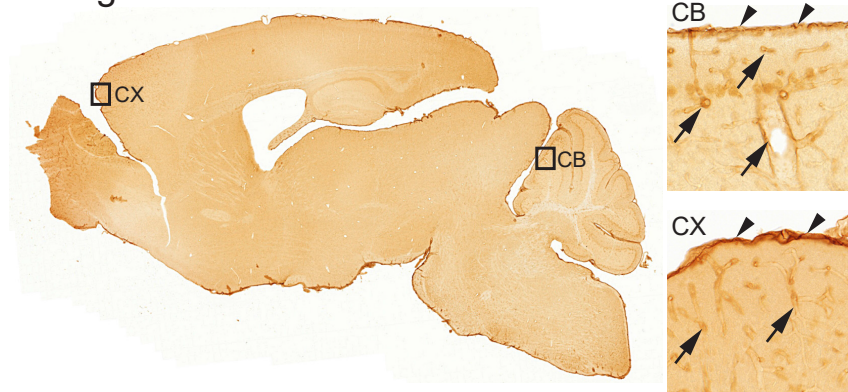

**B: Laminin**

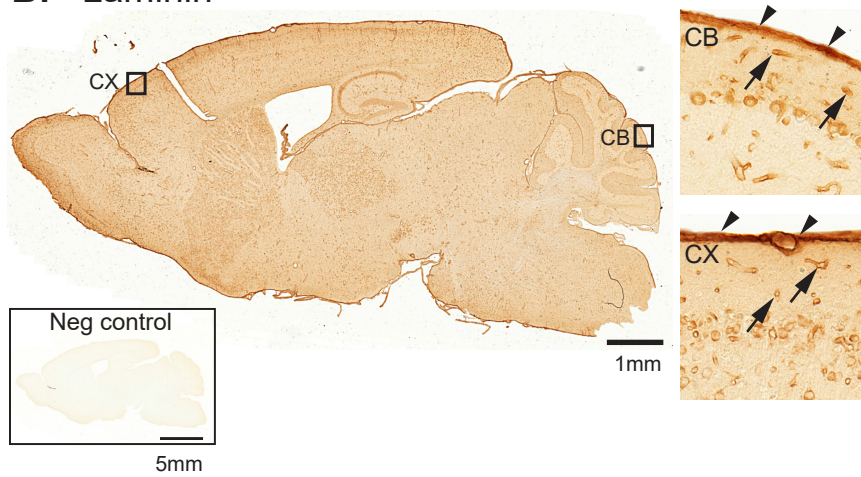

Supplement: Supplementary file 1 — Supplementary figure 1: Immunoperoxidase labelling of agrin (A) and laminin (B) is shown on sagittal sections from an adult mouse brain. Insets show higher magnification from cerebellum (CB) and cortex (CX). Higher magnification images are arbitrarily orientated with brain surface upwards. Both antibodies display a similar staining pattern whereby a distinct brim surrounds all vessels (arrows), consistent with labelling of basal lamina. Staining of brain surface and meningeal covering (insets and overview, arrowheads) is also present. Omitting primary antibodies (left, Neg control, inset) abolishes all labelling. Nuclear and Purkinje cell (inset, CB) staining is interpreted as unspecific. (PDF 9839 kb) [file 429_2020_2036_MOESM1_ESM.pdf]

**A****Agrin**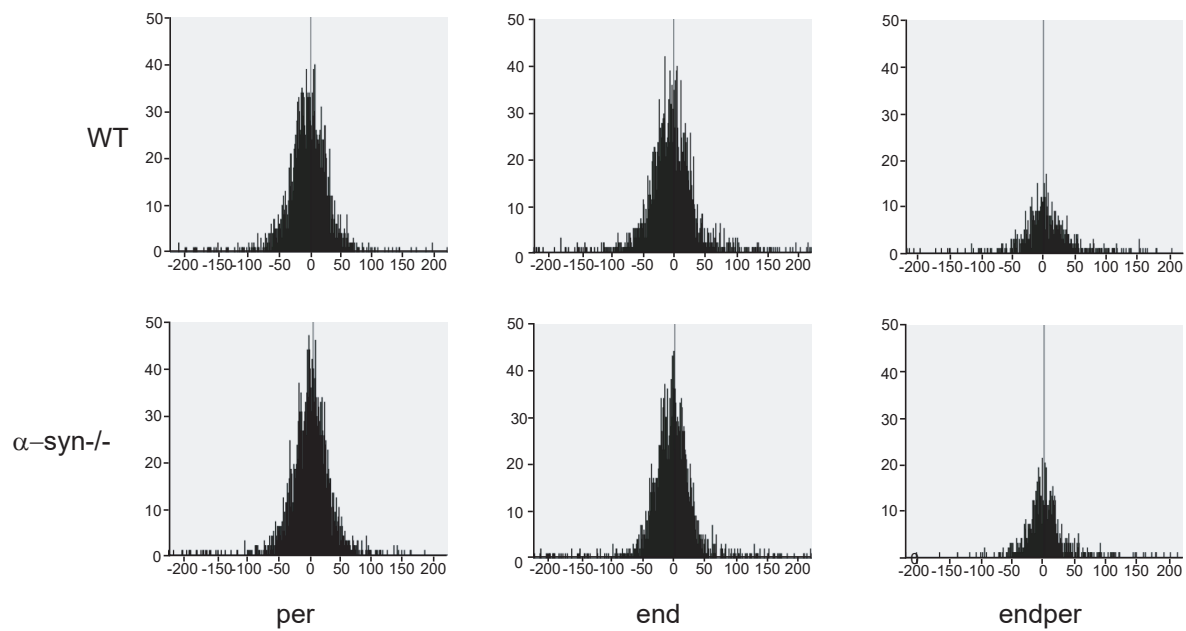**B****Laminin**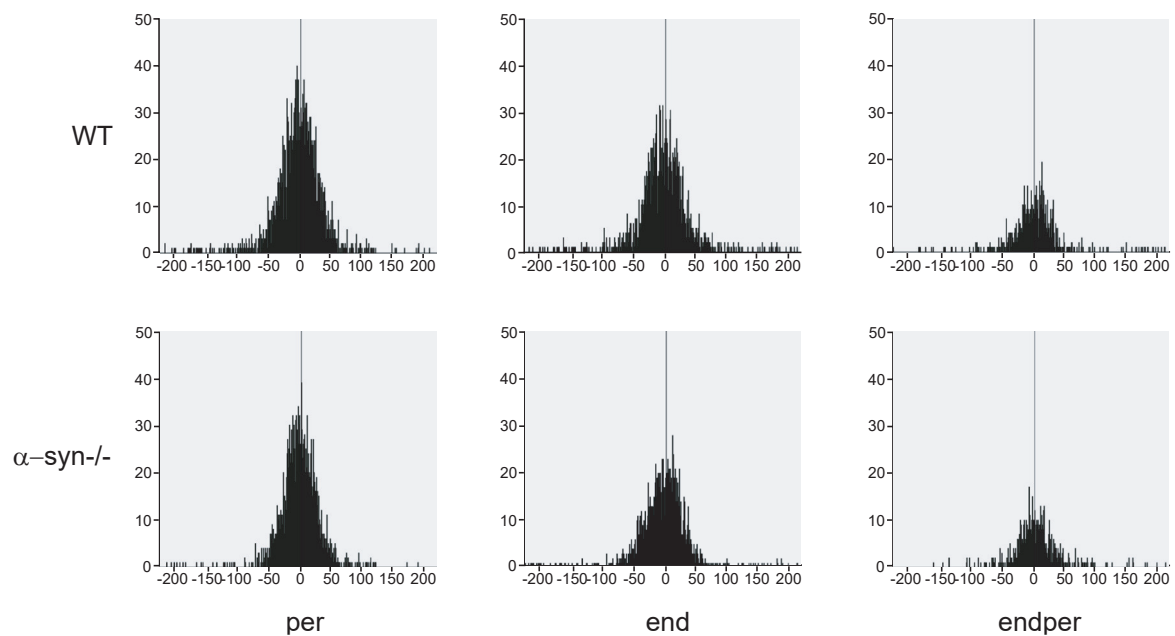

Supplement: Supplementary file 2 — Supplementary figure 2: Histograms of individual basal lamina microdomains per, end and endper are shown. Gold particle distribution along an axis perpendicular to the middle (grey, central line in each histogram) of pericapillary basal lamina is shown for WT and α-Syn-/- genotypes. The capillary lumen is to the right in each graph. Datapoints from CX and CB were pooled. A: Anti-agrin immunogold labelling. B: Anti-laminin immunogold labelling. Gold particles signalling laminin and agrin were normally distributed across the lamina with a distinct peak corresponding to its midline. No difference is seen between WT and α-Syn-/- genotypes nor is any difference in distribution present when comparing individual ROI types with each other. Y-axis shows number of gold particles detected (frequency), X-axis represents distance from midpoint of the basal lamina in nm. (PDF 600 kb) [file 429_2020_2036_MOESM2_ESM.pdf]

**A** Agrin

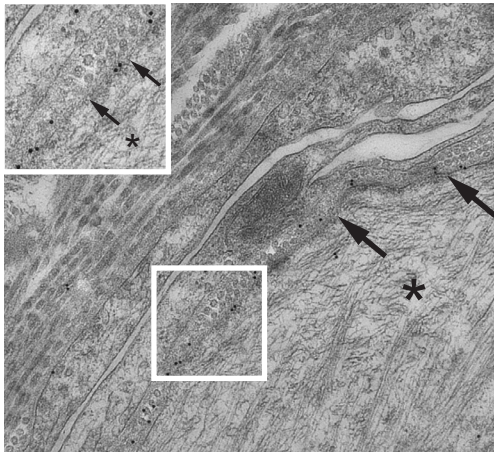

**B** Laminin

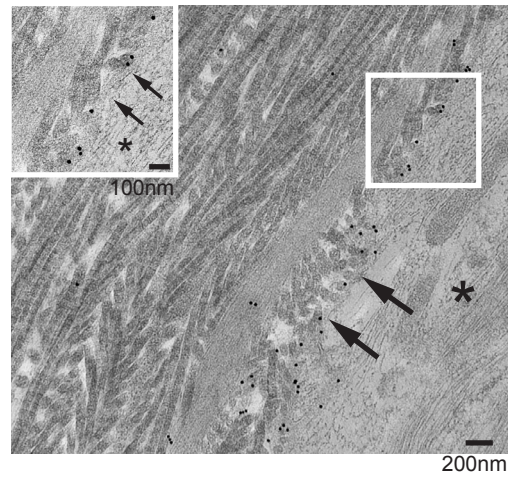

Supplement: Supplementary file 3 — Supplementary figure 3: Immunogold labelling of agrin (A) and anti-laminin (B) adjacent to the subpial glia limitans from optic nerve is shown. The glial limiting membrane consists of astrocytes laden with glial fibrillary acidic protein (annotated with asterisk). Shown in both A and B is the tightly woven meningeal covering with collagen fibres cut in cross-section and in parallel. Gold particles conjugated to both laminin and agrin are concentrated in the basal lamina apposed to the outermost part of the glia limitans (arrows). Insets in both A and B show higher resolution of the region of interest. Scalebar in inset (B) 100 nm, scalebar in picture (B, right) 200 nm. (PDF 1153 kb) [file 429_2020_2036_MOESM3_ESM.pdf]
